# Woody and Herbaceous Species Diversity Respond Differently to Environmental Variables in Semiarid Areas in Ghana

**DOI:** 10.1002/ece3.71253

**Published:** 2025-04-15

**Authors:** Reginald T. Guuroh, Bertrand F. Nero, Fousseni Folega, Kwame A. Oduro, Fred Kalanzi, Gloria K. Adeyiga, Adu‐Gyamfi Asamoah, Mark Appiah, Miracle Obeng, E. Amponsah

**Affiliations:** ^1^ CSIR‐Forestry Research Institute of Ghana Kumasi Ghana; ^2^ Biodiversity Research/Systematic Botany University of Potsdam Potsdam Germany; ^3^ CSIR‐College of Science and Technology (CCST) Accra Ghana; ^4^ DFRT, FRNR, Kwame Nkrumah University of Science and Technology, PMB Kumasi Ghana; ^5^ Géomatique et Modélisation Des Ecosystèmes, Laboratoire de Botanique et Écologie Végétale, Département Botanique, Faculté Des Sciences Université de Lomé Lomé Togo; ^6^ National Forestry Resources Research Institute Kampala Uganda; ^7^ University of Bonn, Institute of Crop Science and Resource Conservation (INRES) Plant Nutrition Bonn Germany; ^8^ MIRO Forestry Company Agogo Ghana

**Keywords:** climate, grazing, land‐use, savanna, species composition, vegetation

## Abstract

In the semiarid areas of Ghana, the interactive effects of various environmental drivers, their relative importance, and their direct and indirect effects on plant species composition and diversity are still poorly understood, hence affecting effective ecosystem management. Using a combined gradient approach, the study investigated the predictors of species diversity of both the woody and the herbaceous layers of a steep land‐use and climatic gradient from the forest‐savanna transition to the Sudan savanna of Ghana. Species richness and the Shannon–Weiner Index are the response variables. Two‐way ANOVA was performed to test the interaction effects of climate and land‐use on species diversity; linear mixed‐effect models were used to test the relationships between multiple environmental variables, and structural equation modelling was used to determine the direct and indirect effects of climate and land‐use on species diversity. We found significant effects of climate and land use, and their interactions on species diversity for both vegetation layers. We also found differential responses of the herbaceous and woody layers to environmental drivers. Land use (Grazing pressure) was the most important predictor of the woody layer while climatic aridity was the most important for the herbaceous layer. Climatic aridity and fire were only directly important for herbaceous vegetation but not the woody layer, although their indirect effects cannot be discounted. For soil properties, organic matter was important for both vegetation layers. *Synthesis:* The marked differences in species composition for various land uses along the climatic gradient imply that climate change will indeed affect vegetation. The observed importance of grazing for all response variables implies that land use could override climate effects and that appropriate land management strategies could mitigate potential negative effects of climate change.

## Graphical abstract

In the drylands of Ghana, the relative importance of various environmental drivers for plant species composition and diversity is still poorly understood and this affects the identification and implementation of appropriate management strategies. Using a combined gradient approach, this study investigated the predictors of species richness and diversity of both the tree and herbaceous layers in the drylands of Ghana. We found clear differences in species composition between land‐use types across the climatic gradient for both the tree and herbaceous layers. We also found differential responses of the herbaceous and tree layers to environmental drivers.

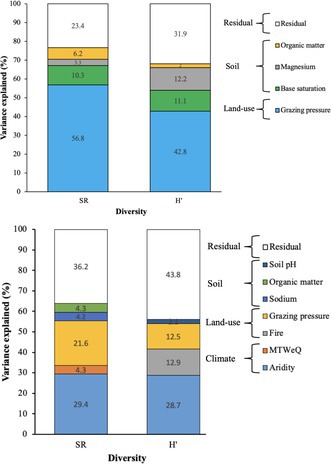

## 1 Introduction

In contemporary ecological research, understanding ecosystem responses to global environmental change is critical (de Bello et al. 2006; Reed et al. 2012). Vegetation composition and diversity play a defining role in ecosystem health and functioning in response to changing environmental conditions (Ruppert et al. 2012). They also underpin the provisioning of ecosystem services that ultimately benefit human well‐being (Allan et al. 2015; Kohler et al. 2017). Ecosystems of all kinds, including forests and savannas, supply various services to society, including provisioning services such as food, water, timber, and fibre; regulating services such as regulation of climate, floods, diseases, wastes, and water quality; cultural services such as recreation, aesthetic enjoyment, and spiritual fulfilment; and supporting services such as soil formation, photosynthesis, and nutrient cycling (MEA 2005). People in developing parts of the world, such as Ghana, depend heavily on natural ecosystems for their survival, thus making them highly vulnerable to environmental changes that have negative consequences on ecosystems (Naah et al. 2017). In the recent past, Africa has been subjected to substantial changes in land use and climate (Yahaya et al. 2024). These changes are projected to continue and to be particularly drastic in West Africa (Knippertz et al. 2015; Yahaya et al. 2024). For example, savannas are predicted to expand in the next few centuries at the expense of tropical forests, mainly as a result of deforestation and human‐caused fires (Alexandre et al. 2011). Deforestation and fires are the most important drivers of vegetation composition, ecosystem functioning, and thus ecosystem services supply (Zerbo et al. 2016; Gessner et al. 2015).

Most previous studies investigated the effects of climate and land use on West African savanna vegetation separately. For instance, the importance of climatic variables governing the distribution of vegetation types across the African continent has been documented (Adejuwon 1997; Swaine et al. 1992), with precipitation regularly cited as the main climate predictor responsible for all vegetation distribution (Sankaran et al. 2005; Bucini and Hanan 2007). In West Africa (and Ghana in particular), vegetation physiognomy and composition display remarkable changes from south to north in response to a corresponding rainfall gradient and rainfall seasonality from the coastal to the Sahelian zone (Bongers et al. 1999; van Rompaey 1993). Ecosystem and biodiversity studies along such climatic gradients may allow one to extrapolate climate change effects using space‐for‐time substitution if spatial trends reflect projected temporal trends in the region (Dunne et al. 2004).

However, some research findings have suggested that rainfall alone cannot be used as a good predictor of vegetation distribution patterns and that other factors should be considered, especially at local/smaller scales (Bongers et al. 1999; Gautier and Spichiger 2004; Taonda et al. 2024). Recent studies of savannas in this region could not find a strong effect of topography and soils on herbaceous vegetation composition and ecosystem functioning (Guuroh 2016). However, Devineau and Fournier (2007) revealed that topsoil characteristics are most important for herbaceous species growth. Similarly, light limitation due to shrub and woody layer, nutrient availability, precipitation, and soil water‐holding capacity regulated herbaceous species richness and species cover (Jakubka et al. 2017). Besides climate and soils, land‐use is another important driver in vegetation composition, with the main land‐use variables in West Africa being grazing, fire, agriculture, and harvesting of both timber and non‐timber forest products (Guuroh 2016; Nacoulma et al. 2011). However, recent studies in the Sahelian‐Sudanian zones found low similarity in species composition among land uses and climate zones (Taonda et al. 2024).

Land‐use changes exert various degrees of influence on ecosystems, from less subtle changes such as a reduction in vegetation cover to drastic and sometimes irreversible changes such as complete shifts in vegetation types and functions, for example, a conversion of forests to savannas or savanna woodlands to open grasslands (Sankaran et al. 2005; Higgins et al. 2010). In fact, land‐use, particularly agricultural practices, can cause drastic declines in plant species (including woody species) diversity (Nacoulma et al. 2011; Balima et al. 2020), reduce woody species cover, and carbon storage potential in savannas (Balima et al. 2020). Agriculture in Ghana, generally practiced in the form of shifting cultivation, transforms forest lands and savannas into mosaic landscapes with croplands, fallows of different ages, and non‐arable savanna sites (Ouédraogo et al. 2015). In addition, natural and anthropogenic fires continue to play key roles in shaping savanna ecosystems. Fires may damage or kill flora and fauna in ecosystems depending on fire regimes, but could also induce the regeneration and growth of some species by breaking the dormancy of the hard‐coated seeds (Dayamba 2010; Yaro et al. 2024). Fires cause a drastic reduction in the stand structure, that is, basal area of some savanna species, for example, *Vitellaria paradoxa* and *Anogeisus leiocarpa* (Amoako and Gambiza 2021). In addition, grazing activities by livestock are mostly extensive and take place almost everywhere. In the past decades, grazing pressure and its consequences have become more pronounced due to the influx of Fulani, who are nomadic herdsmen that move along with large herds of cattle and sometimes smaller herbivores such as sheep (Adriansen 2008).

Despite the foregoing outcomes on drivers of savannas and forest‐savanna transition ecosystems, there are still gaps in our understanding of how different environmental variables affect woody and herbaceous species composition and diversity as well as the relative importance of such variables in the savannas of West Africa. Besides the unilateral focus on either land‐use or climate effects on plant species diversity, the majority of the studies on this subject in West Africa have focused on herbaceous species diversity. Research on the interactive effects of environmental drivers of woody species diversity and composition in the West African savanna and forest transition zone is scanty. The application of space‐for‐time substitution in examining the interactive effects of land‐use, climate, and other environmental variables, although highly recommended (Redlich et al. 2021), has rarely been adopted in the West African savanna to examine woody and herbaceous species diversity and composition. This hampers the formulation and implementation of appropriate land management strategies (Guuroh 2016). Therefore, this study contributes to filling these research gaps. The study examined (i) the interactive effects of climate and land‐use on species composition and diversity of the herbaceous and woody layers, (ii) the relative importance as well as the direct and indirect effects of predictors (i.e., climate, soil, land‐use) (i.e., protection status, grazing pressure, and fire) on the diversity and composition of the herbaceous and woody layers of the semi‐arid areas of Ghana. The study area encompasses semi‐arid and dry sub‐humid areas where climate variability/change, grazing, and fire incidences are especially prominent. Two‐way analyses of variance, linear mixed‐effects models, and structural equation modelling (SEM) have been employed as a powerful integrative framework to address the afore‐stated objectives.

## 2 Materials and Methods

### 2.1 Study Area

**FIGURE 1:**
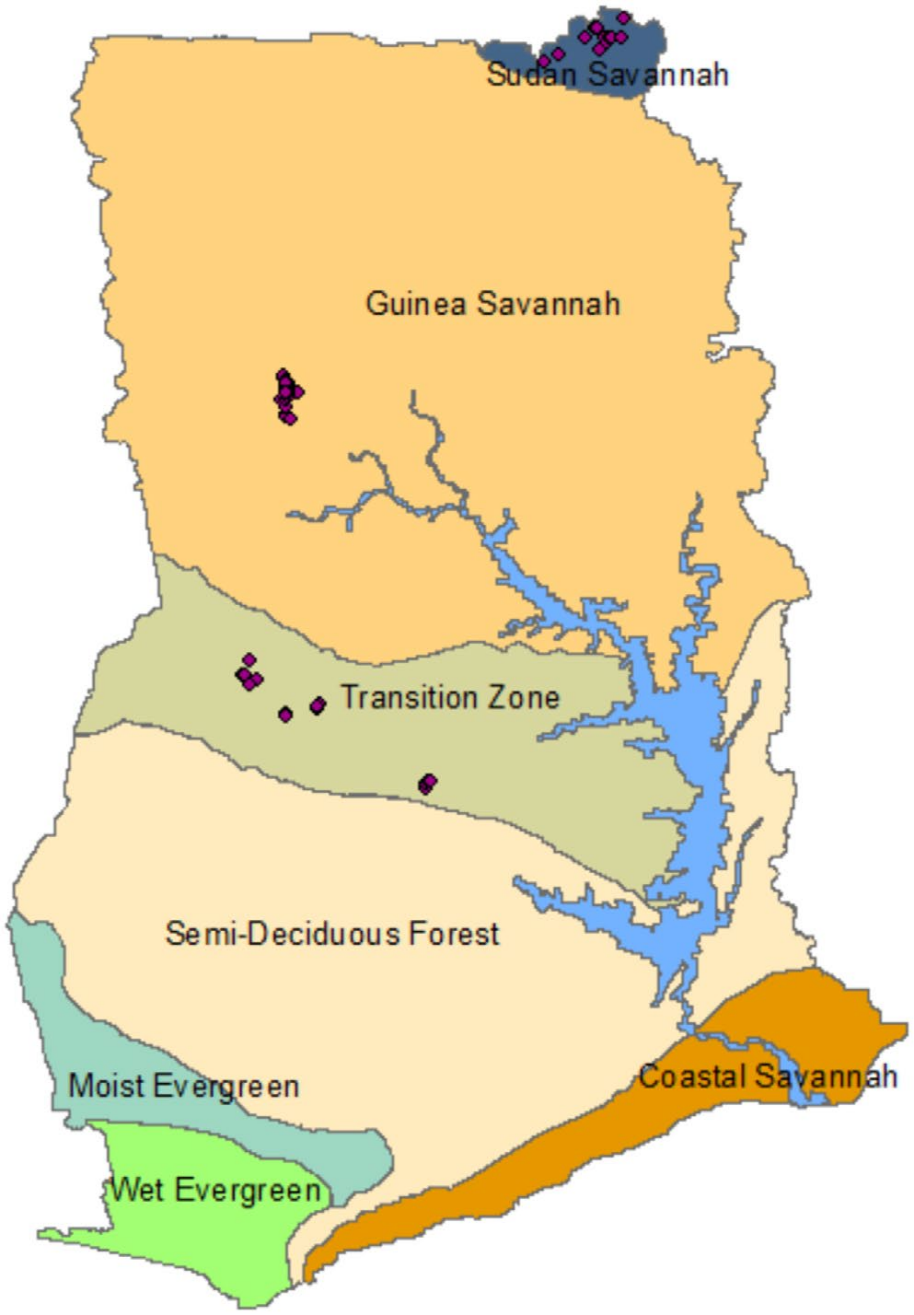
Map of Ghana showing the location of the three ecological zones where the study was conducted (map by Kwadwo Kyenkyehene Kusi). The points or dots on the map represent the sample plot locations.

The study was conducted in three climatic/ecological zones in Ghana: the Forest‐savanna Transition Zone (FSTZ), the Guinea Savanna Zone (GSZ), and the Sudan Savanna Zone (SSZ) (Figure 1). The climate in the study areas is seasonal; in the FSTZ, it is humid to dry sub‐humid; in the GSZ, it is semi‐arid; and in the SSZ, it is semi‐arid to arid (UNEP 1997). Thus, the FSTZ is described in this paper as wet, the GSZ is intermediate, and the SSZ is dry. The soils are generally poor, coarse‐textured, with low water holding capacity, and are highly susceptible to erosion (Callo‐Concha et al. 2012).

The FSTZ stretches over 28% of the land cover of Ghana and, floristically, has a mix of forest and savanna tree species (Attua and Pabi 2013). It is an ecotone characterised by the preponderance of both woody and non‐woody species, shaped by the evolutionary history of changes in abiotic drivers and the coevolution of a wide range of ecological processes (Oliveras and Malhi 2016). In the tropical FSTZ, the predominance of soil moisture availability (rainfall), fire, and grazing determines whether grasses or trees dominate the landscape. The area is believed to have been derived from forest but is now expanding along the forest fringes, with grassland replacing forests (Stanturf et al. 2011). The zone has a bimodal rainfall distribution, receiving up to 1300 mm of annual mean rainfall (MoFA 2016) with a mean annual temperature of 27°C (Asare‐Nuamah and Botchway 2019).

The GSZ is characterised by wooded grassland vegetation. It consists of a ground cover of grasses of varying heights growing under and in between fire‐resistant trees (Asiedu et al. 2017). The less disturbed areas are dominated by relatively short trees with shrub and scrub undergrowth. The GSZ covers about 63% of the total land area of Ghana (Ministry of Environment and Science 2002) with an annual mean temperature of 28.3°C and a unimodal rainfall of 1100 mm/annum which is relatively short in duration and decreasing northwards (MoFA 2016; Asare‐Nuamah and Botchway 2019). This zone has the next highest seasonality index (SI) values after the SSZ, with a shorter dry season of about 5–6 months (Yamba et al. 2023).

The SSZ is located to the north of the Guinea Savanna and is restricted to a small area in the extreme northeast of Ghana, occupying approximately 10,540 km^2^ (Ministry of Environment and Science 2002). The temperatures in this region are generally warm, with an average of 29.2°C and a short rainy season duration with approximately 1000 mm mean annual rainfall (MoFA 2016; Asare‐Nuamah and Botchway 2019). The SSZ has the highest SI values with a markedly seasonal rainfall with a longer drier season of about 7 months (Yamba et al. 2023). The vegetation is made up of isolated and sparse short scrub and grass undergrowth, and trees mainly comprising *Vitellaria paradoxa* , *Diospyros mespiliformis* , *Anogeissus leiocarpa*, *Parkia biglobosa* , and *Tamarindus indica* .

### 2.2 Sampling Design

Field data were collected from all sites during the rainy season (June–September) in 2019. To capture local gradients in land use, stratified sampling was conducted within each rainfall or climate zone, along the climate gradient. The three climate zones considered correspond to the existing ecological zones (FSTZ, GSZ, and SSZ) and were subsequently described as wet, intermediate, and dry areas, respectively. Within each zone, three land use types were delineated, namely protected unburnt areas (PUA), protected burnt areas (PBA), and non‐protected areas (NPA). Tables 1 and 2 below summarise the description of land use types and locations of sampling areas, respectively.

**TABLE 1:** Land‐use types in the study and their description.

Land‐use type	Acronym	Description
Non‐protected area	NPA	Communal area exposed to all types of human land uses without control as opposed to protected areas where human disturbances are not permitted. The NPAs are exposed to fires without any form of control and may be burned more than ones in a season
Protected area	PA	The protected areas refer to areas protected by government law for example, forest reserves and wildlife parks or areas protected by community law for example, sacred groves. Two types (see below) were considered in this study; PBA and PUA
Protected burnt area	PBA	The PBA are areas under protection but experienced (mostly) controlled burning within the last 5 years
Protected unburnt area	PUA	The PUA are areas under protection and not exposed to fires for over 5 years

**TABLE 2:** Location of sampling sites across all climate zones.

Climate zone/land‐use	NPA	PBA	PUA
FSTZ	Off‐reserve areas near Wenchi in Bono Region, Ghana	(1) Tano‐Boase sacred grove (2) Kogyae Strict Nature Reserve Both in Bono Region, Ghana	(1) Buabeng‐Fiema Monkey Sanctuary (2)Nwoase sacred grove Both in Bono Region, Ghana
GSZ	Off‐reserve areas near Damongo and Laribanga, in Savanna Region, Ghana	Mole National Park in Savanna Region, Ghana	Mole National Park in Savanna Region, Ghana
SSZ	Off‐reserve areas near Bawku in Upper East Region, Ghana	(1) Zawse Hills Block 1 (2) Red Volta East Both in Upper East Region, Ghana	(1) Bubungu Extension Forest reserve (2) Upper Tamale Block 5 Forest reserve Both in Upper East Region, Ghana

Identification of land‐use types was done through wide consultation of experts involved in land management such as the Forestry Commission (Forest Services Division and Wildlife Services Division) and traditional leaders (custodians of sacred groves). Besides information by land managers about fire occurrences, we also observed study areas for signs of recent fires e.g., charred or burnt stems of standing trees.

Ten plots of 50 m × 20 m (0.1 ha) were randomly located (at least 1 km from each other) in each land‐use type for the study of woody vegetation. In total, 30 plots were sampled per zone, and 90 plots were used for the study. Two 1 m^2^ sampling quadrats were randomly placed in each 0.1 ha plot (*n* = 180) for herbaceous data collection. This approach ensured that the study captured a representative sample of the woody and herbaceous vegetation across the three ecological zones while accounting for variations in land‐use and rainfall levels.

### 2.3 Data Collection

#### 2.3.1 Climate Variables

For each site, data interpolations on climate variables were obtained from WorldClim (averages over 1950–2000; http://www.worldclim.org/) (Appendix A). The WorldClim dataset was used because of its high spatial resolution of 30 arc‐s or ~1 km, its wide usage worldwide (over 15,000 citations) and quality as well as its use of data from more than 47,000 weather stations from 1950 to 2000 worldwide as input to produce interpolations (Hijmans et al. 2005; Navarro‐Racines et al. 2020). Climatic aridity was quantified based on the UNEP aridity index (AI) as precipitation/potential evapotranspiration as 1‐AI (UNEP 1997; Delgado‐Baquerizo et al. 2013; Ochoa‐Hueso et al. 2016).

#### 2.3.2 Soil Variables

Soil variables (both physical and chemical) were obtained at the plot level (Appendix B). Following FAO (2006), we estimated the cover of surface fragments. To quantify topsoil properties, a composite sample from five soil cores (0–10 cm depth) was collected for each plot (Carter and Gregorich 2006). Samples were homogenised and air‐dried (> 21 days); milled, and only fractions < 2 mm were analysed for their chemical properties at the CSIR‐Soil Research Institute in Ghana.

#### 2.3.3 Land‐Use Variables

In West Africa's savannas, not all officially designated protected areas are actually excluded from use such as grazing by domestic livestock (Ouédraogo et al. 2015; Guuroh et al. 2018). For this reason, this study did not consider a site's protection status as a predictor variable in the LMM, but directly assessed land‐use intensity at the plot level using grazing as a surrogate. Following Linstädter et al. (2014) and Guuroh et al. (2018) physical evidence of grazing pressure (GP) was recorded, ranging from 0 (ungrazed) to 4 (heavily grazed). Physical evidence of grazing was recorded through an expert visual assessment of recent grazing pressure using combined signs of trampling, dung presence, and the removal of standing biomass. Similarly, the study included fire as a categorical variable by differentiating between non‐protected areas, protected burnt areas, and protected unburnt areas. Based on knowledge of the fire behaviour in these areas, these categories were scored as 1 (no fire), 2 (controlled fires) and 3 (regular uncontrolled fires). Ordinal data were treated as quasi‐numerical in further analyses following Guuroh et al. (2018).

#### 2.3.4 Vegetation Properties and Diversity Indices

The species identity, diameter at breast height (dbh or stem diameter at 1.3 m from the ground line) and canopy height of all adult trees and shrubs with a stem diameter (dbh) ≥ 5 cm were recorded in each 0.1 ha plot. Taxonomic Diversity (TD) for both woody and herbaceous layers was quantified using species richness (SR) and the Shannon–Wiener Index (H′) (hereafter Shannon index). Species richness refers to the number of individual species in a community (Collwell et al. 2012) and was determined as such for each plot. The Shannon–Wiener Species Diversity Index is calculated using the equation below (Equation 1), which is the sum of the proportion of species i multiplied by the natural log of the proportion for each species (Collwell et al. 2012). The higher the number, the higher the species diversity.

(1) $$H^{\prime }=-\sum_{i=1}^{S}p_{i}\log_{b}p_{i}$$

Where H' = Shannon diversity index, S = species richness, pi = proportion of individuals belonging to the ith species, and b = base of logarithm.

### 2.4 Data Analysis

To evaluate the joint effects of climate and land‐use on SR and H', we first used two‐way ANOVA. The species diversity data were normally distributed (Shapiro–Wilk test, W = 0.97, *p* = 0.0823) and the homogeneity of variances assumption was satisfied (Levene's test, *F*‐value = 1.28; *p* = 0.2649). Significant main and interaction effects were subjected to Tukey multiple comparison tests. In a second step, we selected environmental variables as potential predictors of diversity, explored their relationship with diversity indices, and then calculated the relative importance of the model predictors. Finally, to disentangle the joint effects of climate and land‐use drivers and quantify their direct, indirect, and combined effects on species diversity, Partial Least Squares Path Modelling (PLS‐PM; Tenenhaus et al. (2005)) was used. This is a non‐parametric, composite‐based type of structural equation modelling (SEM) which uses multivariate methods that allow the linkage of measurable attributes (i.e., indicator variables) to underlying hypotheses or theoretical concepts by means of (unobserved) latent variables (LV). The LVs are constructs of predictors which are merged, based on correlations, into a single robust variable or component (i.e., similar to the components of PCA or CCA, but in a supervised manner).

#### 2.4.1 Selection of Environmental Variables

Separate principal component analyses (PCAs) were performed for three variable sets: (i) climate, (ii) soil chemical properties, and (iii) Soil physical properties (Appendix B). We identified variables highly loading (≥ 0.8) on principal components (PCs) with eigenvalues > 1 to reduce collinearity within predictor sets. Given their ordinal or categorical nature, site, grazing pressure (GP) and fire occurrence could not be included in PCAs and were selected due to their prevalent importance. The multi‐collinearity of selected variables was checked using Pearson's correlation and variance inflation factors (Appendix C).

#### 2.4.2 Exploring the Relation Between Environmental Variables and Plant Species Diversity

To assess the effects of environmental variables on diversity, linear mixed‐effects models (LMM) were used. Before modelling, all explanatory variables were standardised by scaling and centering. Initially, full LMMs—including all selected variables as fixed effects—were established for each of the response variables; ‘site’ was included as a random‐intercept term. Initial full models were subjected to AIC‐based model selection using restricted maximum likelihood estimation. LMMs were calculated using the lme4 package (in version 3.2.2) for R (Bates et al. 2015). To estimate the variance explained by fixed and random effects in final models, we used the method proposed by Nakagawa and Schielzeth (2013) to obtain marginal and conditional *R* ^2^ (MR^2^ and CR^2^, respectively). MR^2^ is the proportion of explained variance by fixed effects, and CR^2^ is the proportion explained by fixed plus random effects (Ruppert et al. 2015). For each of the woody and herbaceous layers, variance explained by random effects was calculated as CR^2^ minus MR^2^.

Final LMM models were further explored using type III ANOVA. We estimated the importance of each individual predictor by calculating classical eta‐squared values as an effect size metric. The eta‐squared value for a given predictor reflects the proportion of total explained variance in the dependent variable that is associated with this very predictor under the given model (Cohen 1973). To validate and estimate uncertainty in the standard error (SE) of each model parameter, we bootstrapped our final models (10,000 repetitions with replacement). We also calculated the relative bias in SE for model parameters by comparing the bootstrap estimates and our final LMM, following Equation (2) (Thai et al. 2013):

(2) $$\text{RBias}=\frac{{\mathrm{SE}}_{\text{Boot}}-{\mathrm{SE}}_{\mathrm{LMM}}}{{\mathrm{SE}}_{\mathrm{LMM}}}\times 100$$

Where RBias is the relative bias;

${\mathrm{SE}}_{\text{Boot}}$ = Bootstrap standard errors averaged over 10,000 runs.

${\mathrm{SE}}_{\mathrm{LMM}}$ = Final LMMs' standard errors.

Following Thai et al. (2013), we classified model predictors as unbiased (RBias < ± 5%); moderately biased (±5%–10%); and strongly biased (> ±10%). Bootstrapping was performed with the boot package (in version 3.2.2) for R. Statistical assumptions were explored visually as proposed by Zuur et al. (2010).

### 2.5 Structural Equation Modelling

Structural equation modelling (SEM), a powerful multivariate statistical technique, was adopted in testing the direct and indirect effect of various climate, land‐use, and soil variables on woody and herbaceous species diversity in the semi‐arid areas of Ghana. SEM uniquely tests direct and indirect effects of presupposed causal relationships (Fan et al. 2016; Wondimu et al. 2021). One major advantage of SEM is its ability to visualise data and hypotheses in a graphic model (Fan et al. 2016). To detect the effects of abiotic processes (climate and soil properties) and land‐use activities on species diversity, we linked the measures of anthropogenic and abiotic environmental parameters to the measures of diversity (species richness and Shannon diversity index). One structural equation model specified represents partial mediation, which postulates that there are both direct and indirect effects of land use (grazing pressure, fire incidences and protection status), climate (e.g., climatic aridity, temperature etc.), soil properties (soil organic matter/carbon, base cation concentrations, pH, etc.) on diversity parameters of each of the woody and herbaceous vegetation layers in the three ecological zones. For the herbaceous SEM model, stand structure/density (represented by number of stems and basal area) was included, in addition to the environmental variables selected for the LMMs due to the fact that stand structure attributes have the potential to affect herbaceous vegetation presence, composition, and diversity. Because of the multiple measures of each predictor variable, stepwise selection techniques were used to select the most relevant soil, climate, land use, and climate‐land‐use interaction effects on diversity. The SEM analysis was performed using the Partial Least Squares Path Modelling (PLSPM) package in R (Sanchez 2013; Esposito Vinzi et al. 2010; Tenenhaus et al. 2005). The quality of the SEM model is evaluated by examining three indices: the *R* ^2^ determination coefficients, the redundancy index, and the goodness of fit index (GoF) (Sanchez 2013). Of these indices, the GoF (a pseudo‐goodness of fit measure) is typically used as a global criterion to evaluate the performance of the model in both the inner and the outer models by taking into account the communality and the *R* ^2^ determination coefficients. The standardised coefficients were used to make direct comparisons across paths. All analyses were conducted using the statistical software R in version 4.4.1 (R Core Team 2024).

## 3 Results and Discussion

### 3.1 Species Diversity of the Woody Layer

The study recorded a total of 144 woody plant species from 37 families (Appendix D) with the woody layer being dominated by Leguminosae (22.5%) and Combretaceae (8.1%). While eight species were ubiquitous, 17 were unique to the dry Sudan savanna zone (SSZ), 23 to the intermediate guinea savanna zone (GSZ) and 75 to the wet forest‐savanna transition zone (FSTZ). The three most abundant species (based on occurrences/abundance in plots) were *Vitellaria paradoxa* (44), *Terminalia avicenoides* (42) and *Anogeisus leiocarpa* (30) (Appendix D). The study also recorded several rare species (occurred only once or twice in all study plots): up to 41 species were recorded only once in all the ninety plots surveyed (see Appendix D). Examples include *Acacia dudgeoni*, *Adansonia digitata* , *Albizia ferruginea* , *Annona senegalensis* , *Parkia bicolor* among others. Their rarity might mean that they are gradually being lost in the landscape and require interventions to prevent them from going completely extinct.

### 3.2 Interactive Effects of Climate and Land‐Use on Woody Diversity

The study found significant differences in species richness (SR) (*p* < 0.0001) and Shannon index (H′) (*p* < 0.0001) between climate zones, land uses, and their interaction (Table 1 and Appendix E). Both SR and H′ showed an increasing trend along the climatic gradient from the dry SSZ to the wet FSTZ (Table 3). The results can be attributed to the variations in environmental conditions (such as rainfall) and soil properties in the various ecological zones. This is supported by Bognounou et al. (2009) and Schmidt et al. (2005), who reported a positive relation between rainfall and woody species richness as well as overall species richness, respectively. Similarly, high species diversity (richness and Shannon) in protected unburnt areas compared to the burnt areas in the dry (SSZ) and wet (FSTZ) areas aligns with other previous studies (das Graças Costa et al. 2022). The occurrence of a significant difference in species richness but a lack of a significant difference in H′ between FSTZ and the GSZ implies that the two ecological zones were different in their number of species but not in the evenness of species composition.

**TABLE 3:** Mean species richness and Shannon–Wiener index of the woody layer along the climatic gradient and land‐use types.

Land‐use/climate zone	Species richness			Shannon–Weiner Index		
	Dry	Intermediate	Wet	Dry	Intermediate	Wet
NPA	3.40 ± 0.56_b_^A^	6.90 ± 0.89_a_^A^	5.70 ± 1.14_a_^A^	0.90 ± 0.20_b_^X^	1.69 ± 0.16_a_^Z^	1.46 ± 0.21_a_^ZX^
PBA	3.40 ± 0.79_b_^B^	8.200 ± 0.49_a_^A^	10.60 ± 1.08_b_^A^	0.90 ± 0.24_b_^X^	1.94 ± 0.09_a_^Z^	2.16 ± 0.10_a_^Z^
PUA	6.90 ± 0.59_a_^B^	8.700 ± 0.87_a_^B^	17.30 ± 1.32_c_^A^	1.82 ± 0.09_a_^X^	1.94 ± 0.08_a_^ZX^	2.53 ± 0.10_b_^Z^

*Note:* Means within the same column followed by the same lowercase subscript letter are not statistically different at alpha = 0.05. Also, means within the same row for each diversity parameter followed by the same superscript capital letter are not statistically different at alpha = 0.05.

Abbreviations: NP, non‐protected burnt area; PBA, protected burnt area; PUA, protected unburnt area.

The study also found a positive effect of land‐use on SR (*p* = < 0.0001) and H′ (*p* = < 0.0001) with the protected unburnt areas (PUA) having the highest SR and H′ within ecological zones, followed by protected burnt areas (PBA) while the lowest was recorded in non‐protected areas (NPA) (Table 3 and Appendix E). In the case of the dry SSZ, the NPA and the PBA were similar but a higher SR was recorded in the PUA (Table 3). The findings suggest that land‐use types (i.e., protection status) had effects on the composition and diversity of woody species in different climate zones (Zerbo et al. 2016) which supports the legacy effect. The findings further confirm that both climate and land‐use (disturbance) together with the soil characteristics interactively shape the diversity and composition of the woody vegetation in the three ecological zones.

**FIGURE 2:**
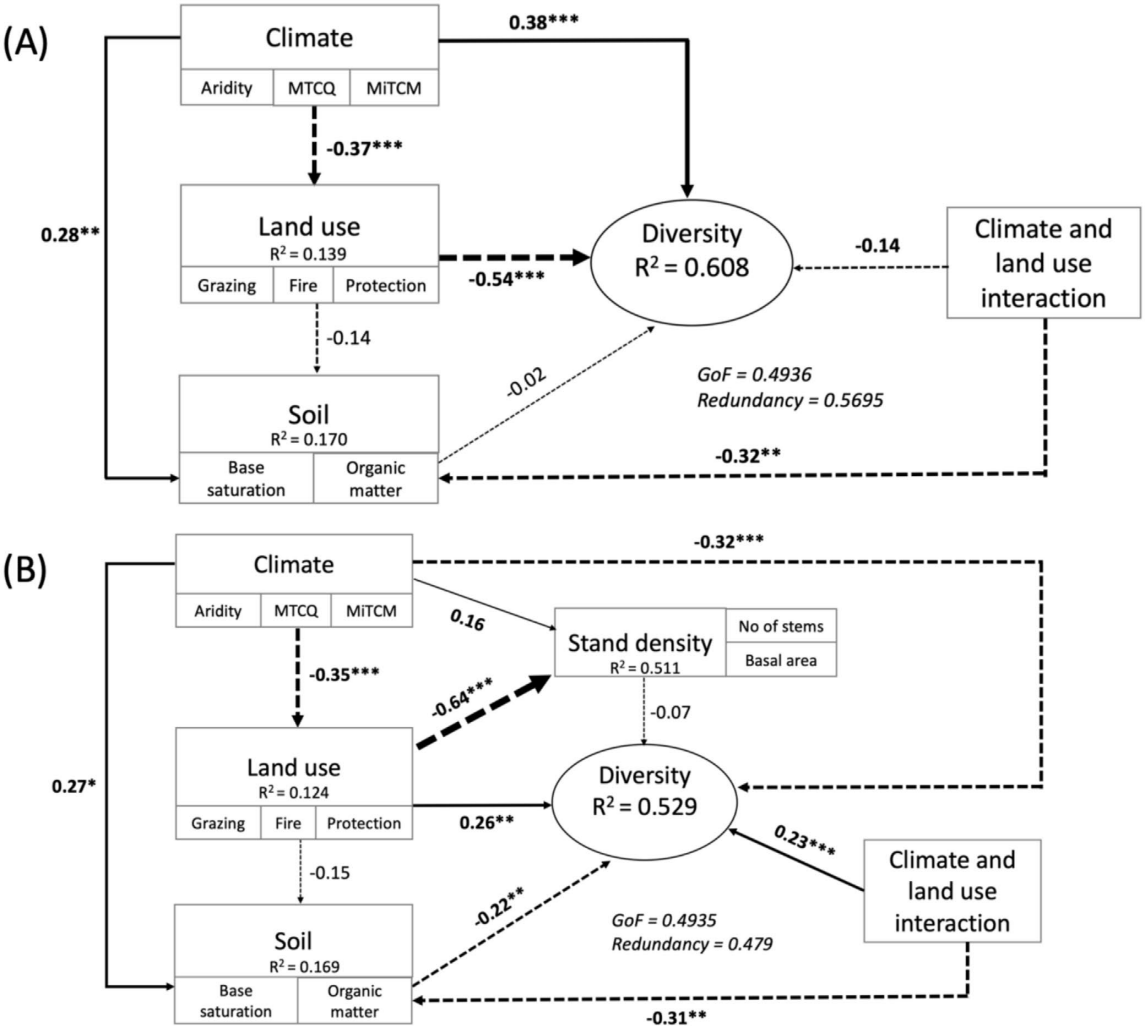
Structural equation model showing the effects of climate, land‐use, soil and interaction of climate and land‐use on species diversity of (A) woody layer and (B) herbaceous layer. Solid black lines represent positive linear associations, while dashed lines indicate negative linear associations. The width of the arrows is proportional to the strength of the relationship. ***p < 0.001, **p < 0.01, *p < 0.05. Aridity = climatic aridity of the site calculated as 1‐UNEP aridity index; MTCQ = mean temperature of the coldest quarter; MiTCM = minimum temperature of the coldest month.

Results from the two‐way ANOVA indicate that the response of SR and H' of the woody layer to land‐use differed between the different climate zones (significant land‐use × climate zone interaction) (Table 3 and Appendix E). Also, the SEM analysis revealed similar results to those of the ANOVA and LMM: Climate, land‐use, and their interaction had significant effects on the diversity of the woody layer (Figure 2A). The SEM model for the woody layer had a high coefficient of determination (*R* ^2^) of 0.608, a redundancy value of 0.5695, and a goodness of fit (GoF) of 0.4936. These values indicate that the variance in diversity explained by climate, land‐use, and soil is 60.8% (using the *R* ^2^), 56.95% (using the redundancy) and 49.36% (using the GoF) (Figure 2A). While climate exerted a positive effect, land‐use, soil, and the interaction between climate and land‐use all showed negative effects on diversity (Figure 2A).

Our results showing less distinction in SR between the various land‐use types in the GSZ and SSZ than in the FSTZ (Table 3) may be due to weaker protection status in the savanna areas compared with the transition area or greater recovery rates of the vegetation in response to favorable climatic conditions (i.e., greater moisture content). Land‐use is well known as a driver of species diversity, with protected areas usually having higher diversity than non‐protected areas (Taonda et al. 2024; Zerbo et al. 2016). Heavy dependence on natural ecosystems in the savannas for charcoal, shifting cultivation, and grazing (Andoh et al. 2024) has resulted in encroachment into protected areas; hence, it was challenging, especially in the SSZ, to find properly protected plots of good condition, which may explain the fewer species recorded. In the GSZ, the protected area used in this study was a wildlife park, and therefore, although it was excluded from grazing by domestic herbivores, it was nonetheless grazed by wild animals. Also, the distinction in bush fire occurrences in the wildlife park was not clear, as park management had some difficulty in confirming fire incidences over the past 5 years before the study. These might have affected species richness and diversity in the Mole Park. It is also likely the species recovered better and faster from grazing and fire pressures in the FSTZ due to greater soil moisture contents and precipitation (Lin et al. 2022).

### 3.3 Relative Importance, Direct and Indirect Effects of Variables on Woody Layer Diversity

The LMM model revealed that 76.5% of the variation in SR and 68.1% of the variation in Shannon–Wiener Index (H′) could be explained by the predictors. Interestingly, the random factor ‘site’ (calculated as conditional *R* ^2^—CR^2^ minus marginal *R* ^2^—MR^2^) had a higher importance for H' (14.5%) than for SR (1.8%) (Appendix F). However, the reliability of both models was limited, as determined by bootstrapping and the calculation of relative bias: both having a ratio of three biased predictors to one unbiased predictor (Table 3).

**FIGURE 3:**
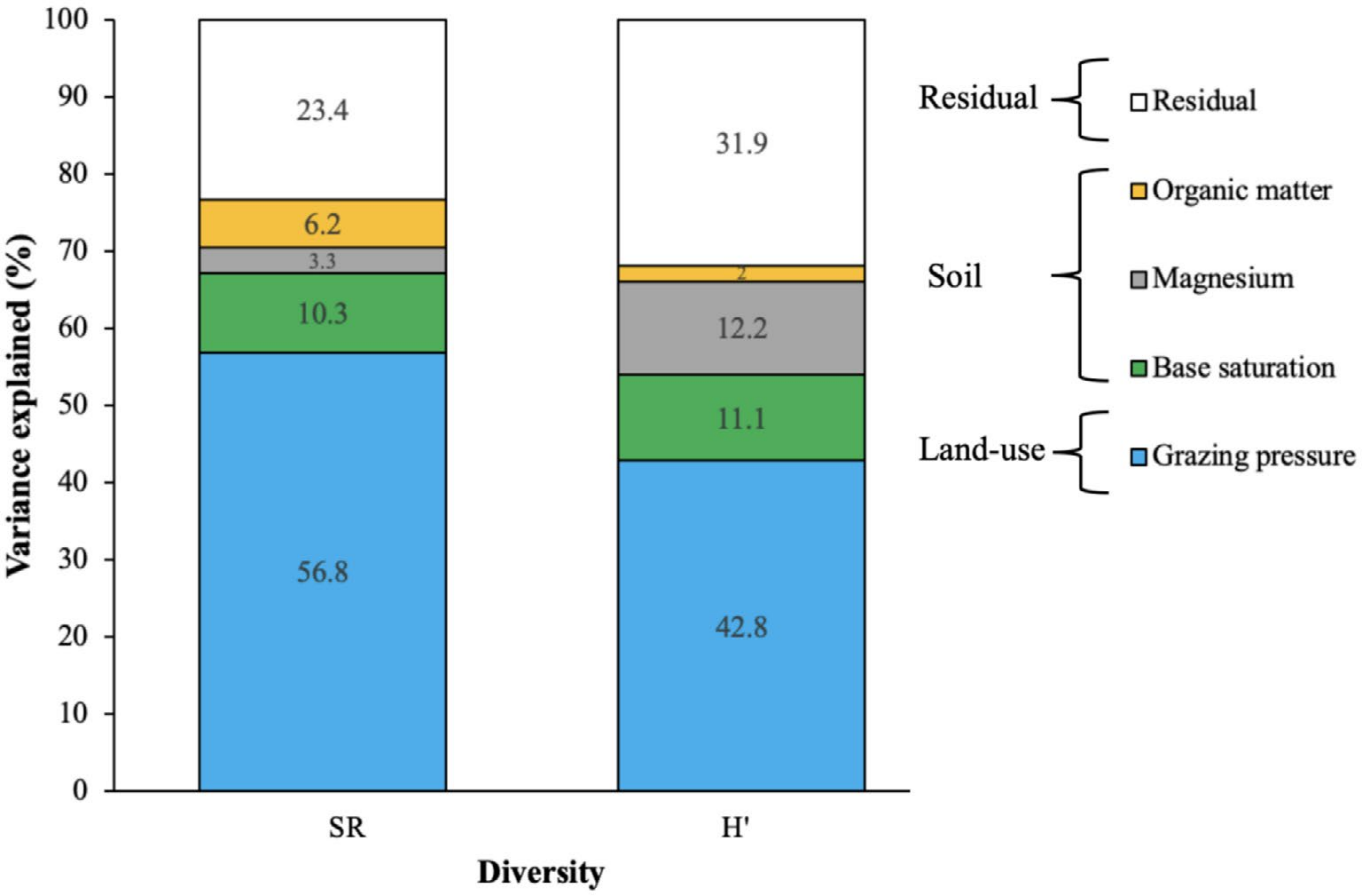
Variance explained by climate, soil, and land‐use predictors in the linear mixed‐effect model. For each response variable, bars denote the proportion of variance explained by significant predictors, calculated as classical eta squared. Unexplained variances are included as residuals. H', Shannon–Wiener Index; SR, species richness.

The main predictors of SR were grazing (56.7%), base saturation (10.3%), and organic matter (6.2%) (Figure 3). Grazing had a negative effect on SR, while base saturation and organic matter had positive effects. The main predictors of H' were grazing (42.8%), magnesium (12.2%) and base saturation (11.1%) (Figure 3). Grazing and magnesium had negative effects, while base saturation had a positive effect.

Results of the SEM corroborated the LMM findings mainly for climate and land‐use, which showed significant positive direct effects on woody diversity (Figure 2A; Appendix G). However, SEM results showed only non‐significant negative effects of soil on diversity (Figure 2A). This indicates that soil (SOM) marginally reduces the species diversity of the woody layer in these climate zones but is not a major driver or determinant of woody layer species diversity.

The study found that overall, the FSTZ had the highest H' (2.05 ± 0.63) followed by the GSZ (1.86 ± 0.38) and the SSZ (1.20 ± 0.72). Even within land‐use types, the more mesic FSTZ recorded the highest H' compared to the drier climate zones (Table 3). These results indicate a higher SR and species evenness in the mesic FSTZ than in the relatively drier savanna zones. Within the savannah of West Africa, declines in species richness from wet to dry precipitation gradients are not uncommon (Jakubka et al. 2017). With an equivalent amount of stress, high moisture contents result in a wide range of microsites which favour a greater number of woody species in the FSTZ compared to the relatively drier climate zones. The negative effect of grazing (as a proxy of land‐use pressure) on SR and H' of the woody layer suggests that anthropogenic disturbances negatively affect the woody layer. This finding is consistent with previous studies by Par'e et al. (2010), Kabor'e et al. (2014) and Ouédraogo et al. (2015) which also reported negative effects of anthropogenic disturbances on woody vegetation in the West African savannas. The soil effects on woody diversity from LMM are consistent with studies highlighting base saturation as a key predictor of woody species richness of Savannas (Veríssimo et al. 2024). The observed importance of soil properties in this study provides evidence that soil also plays a role in influencing woody species diversity, although not at the same magnitude as land use. Overall, the findings highlight the importance of considering land‐use and soil properties in the management and conservation of woody vegetation in the West African savannas.

It may be deduced that local (plot‐level) drivers such as land‐use (disturbance) and soil play a critical role in species diversity; as also found by previous studies (Guuroh et al. 2018). Although climatic variables such as aridity and mean temperature of the wettest quarter were included in the full linear mixed effect model, none of them were retained in the final model. This may not necessarily mean that climate plays no role in species diversity (see SEM results that capture effects of climate, Figure 2A) but rather that local conditions may override large‐scale variables like rainfall which also tend to have more long‐term and less direct effects than local changes. A previous study in the same region found strong indirect effects of protection and climatic aridity on forage supply and also reported that these indirect effects were even stronger than their direct effects (Ferner et al. 2018).

### 3.4 Species Diversity of Herbaceous Layer

The herbaceous layer in this study was quite diverse, with a total of 103 plant species (Appendix H). Out of these, 64 species were recorded in the dry SSZ, 55 in the wet FSTZ, and 54 in the intermediate GSZ. The study recorded 18 ubiquitous species; 23 species were unique to the SSZ, 14 were unique to the GSZ, and 20 were unique to the FSTZ.

### 3.5 Interactive Effects of Climate and Land‐Use on Herbaceous Diversity

ANOVA results show significant differences in species richness (SR) (*F*_2,81_ = 32.74, *p* < 0.0001) and Shannon Weiner index (H′) (*F*_2,81_ = 24.32, *p* < 0.0001) between climate zones (Table 3 and Appendix I). Both SR and H′ showed decreasing trends along the climatic gradient from the dry SSZ to the wet FSTZ (Table 3). In savannas, herbaceous species richness and diversity often increase with increasing precipitation (Zerbo et al. 2016; Nacoulma et al. 2011). This belies the trends observed in the dry SSZ to the wet FSTZ in the current study, which can be due to local disturbance and other land‐use/site pressures. For instance, intense grazing and fire pressures coupled with greater tree density and crown cover in the wet FSTZ relative to the drier guinea and Sahel savanna zones could account for these contrasting trends in SR and H′ across the climate zones. Indeed, the interaction effects in the ANOVA and the indirect effects of land‐use on woody layer stand structural effects (albeit small i.e., −0.07) on herbaceous species diversity categorically illustrate the afore explanations (Figures 2B and 5). Despite showing a low insignificant relationship between (woody layer) stand density and herbaceous species diversity, the role of woody layer stand density on herbaceous species diversity is well documented in the literature (from thinning studies) across the globe. For example, Hare et al. (2021) reported that grass species composition and biomass increased with increasing intensity of thinning, while Riginos and Grace (2021) equally found marked variations in herbaceous species composition with tree density in Kenya. Also, an increase in herbaceous vegetation density was recorded in South Korea, following thinning of *Chamaecyparis obtusa* stands (Sang‐Hyun et al. 2018). In South Africa, thinning of *Colophospermum mopane* trees increased the grass species (Smit and Rethman 1999). Again, it was found in Taiwan that thinning of the *Cryptomeria japonica* forest between 25% and 50% led to increased plant diversity and richness (Tsai et al. 2018). This relationship may be ascribed to reduced competition and higher sunlight availability for understory plants with reduced woody density and cover (Hare et al. 2021; Sang‐Hyun et al. 2018). In general, because the removals of woody species by mechanical thinning are typically sparsely distributed, thinning may increase light variability to the understory through the creation of light patches in thinned areas while leaving light levels relatively unaffected at unthinned sites (Chiang et al. 2012).

The relationships between SR and land‐use type for each ecological zone are presented in Table 3: in the intermediate GSZ, SR was highest in PUA and lowest in the NPA while the opposite trend was observed in the wet FSTZ with the dry SSZ exhibiting a convex curve that is high in communal area, decrease in PBA and an increase again in the PUA. Meanwhile, the study also found a significant positive effect of land‐use on SR (*F*_2,81_ = 21.42, *p* = < 0.0001) and H′ (*F*_2,81_ = 14.84, *p* = < 0.0001) (Table 3 and Appendix I). Our finding that land‐use (grazing and fire occurrences) and soil significantly affect herbaceous species diversity aligns with many previous studies (Nacoulma et al. 2011; Zerbo et al. 2016; Jakubka et al. 2017).

Results from the two‐way ANOVA indicate that the response of SR and H' of the herbaceous layer to land‐use was different between the three climate zones (significant land‐use × climate zone interaction) (Table 5 and Appendix I). Also, the SEM analysis revealed similar results: Climate, land‐use, and their interaction had significant effects on the diversity of the herbaceous layer (Figure 2B). The SEM model for the herbaceous layer had a coefficient of determination *R* ^2^ of 0.529, a redundancy value of 0.479, and a goodness of fit (GoF) of 0.4935. These values indicate that the variance in diversity explained by climate, land‐use, and soil is 52.9% (using the *R* ^2^), 47.9% (using the redundancy) and 49.35% (using the GoF). Unlike the woody layer, climate, soil, and the interaction between climate and land‐use exerted negative effects on diversity, while land‐use showed a positive effect on diversity (Figure 2B).

The LMM results showed explained variance of 63.8% in SR and 56.2% in H′. The random factor ‘site’ (calculated as CR^2^ minus MR^2^) was an important factor, with a higher explained variance for SR (21.8%) than H' (10.3%) (Appendix F). However, after bootstrapping and calculating the relative bias, both models showed limited reliability with all predictors of the SR model being biased and a ratio of three biased predictors to one unbiased predictor for the H′ model (Table 4).

**TABLE 4:** Relative bias of standard errors (SEs) for all significant predictors of diversity.

Predictor	Relative bias of SE (%) for woody layer		Relative bias of SE (%) for herbaceous layer	
	SR	H′	SR	H′
Base saturation	−11.69	57.29		
Grazing pressure	(−2.36)	−7.78	−7.06	(1.88)
Magnesium	−15.66	(−3.45)		
Organic matter	−22.34	37.19	−14.81	
Aridity			−30.32	−17.85
Fire				65.59
pH				−6.61
Mean temperature of the wettest quarter			−28.33	
Soil silt content			−11.79	
Sodium			−27.39	

*Note:* Levels of bias are: Unbiased, with relative bias < ±5% (given in brackets); moderately biased, with relative bias from ±5% to ±10%; and strongly biased, with relative bias > ±10% (given in bold). Empty cells indicate that a potential predictor was not retained in the final model of the respective response variable.

Abbreviations: H′, Shannon–Wiener index; SR, species richness.

The main predictors of SR were aridity (29.4%) and grazing (21.6%), both of which had positive effects. Similarly, the predictors of H′ were aridity (28.7%), fire (12.9%), and grazing (12.5%), all of which had positive effects (Figure 5). Our findings suggest that aridity, fire, and grazing are important factors that influence species richness and diversity in the studied ecosystems, with all exerting positive effects on H′.

**FIGURE 4:**
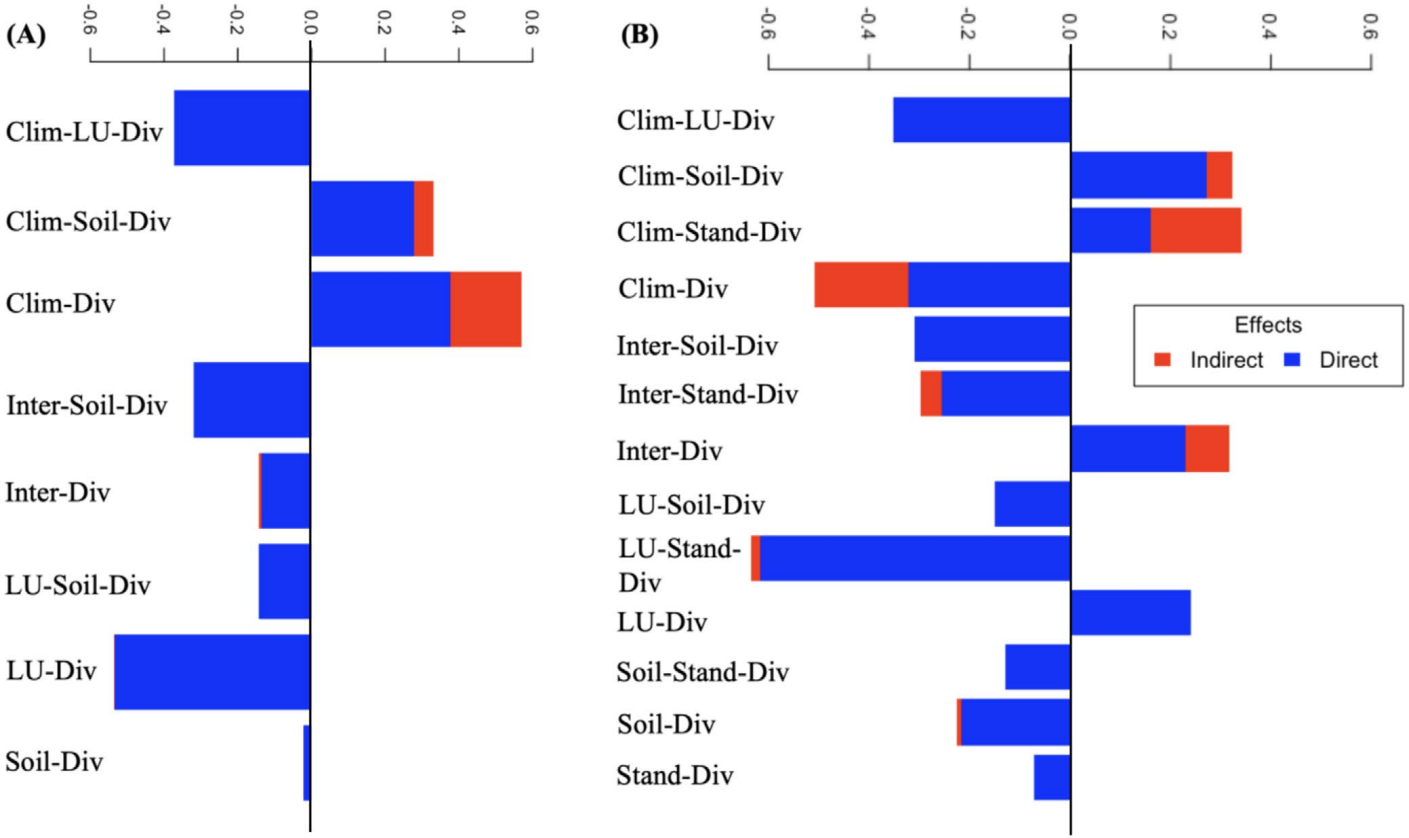
Structural equation model results showing direct and indirect effects of environmental variables on species diversity of (A) diversity of the woody layer, (B) diversity of the herbaceous layer. Clim, climate; Div, diversity; LU, land use; Stand, stand structure/density of the woody layer.

SEM results corroborated the LMM findings showing significant effects of climate, land‐use, and soil on herbaceous layer diversity (Figure 2B). SEM results also revealed that climate and land use exert both direct and indirect effects on herbaceous species diversity (Figure 4 and Appendix GA).

**FIGURE 5:**
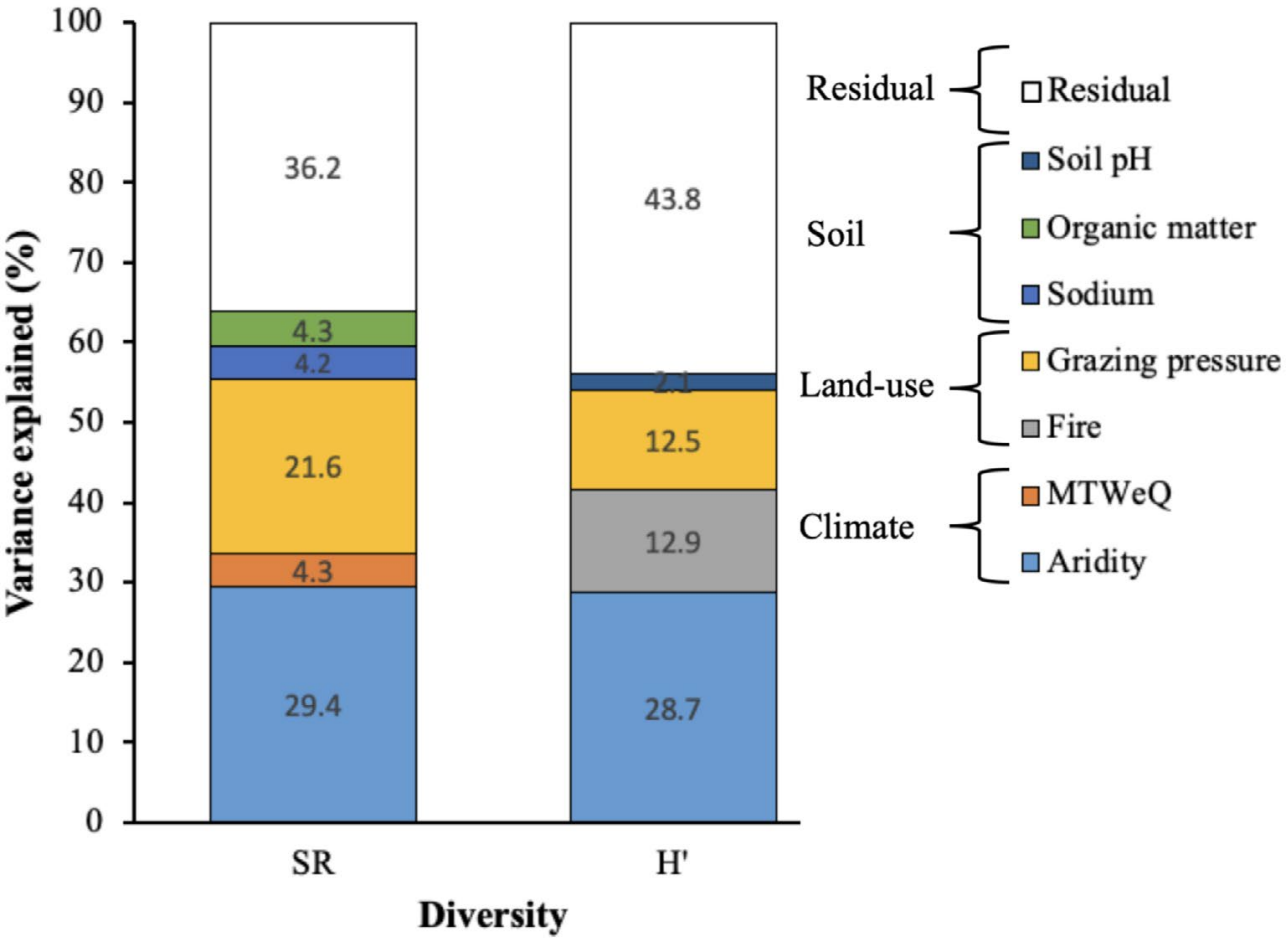
Variance explained by climate, soil, and land‐use predictors in the linear mixed‐effect model. For each response variable, bars denote the proportion of variance explained by significant predictors, calculated as classical eta squared. Unexplained variances are included as residuals. H′, Shannon–Wiener index; MTWeQ, mean temperature of the wettest quarter; SR, species richness.

Our results of decreases in SR and H' with increasing rainfall (from the dry SSZ to the wet FSTZ) contrast findings of other researchers such as Zerbo et al. (2016) and de Bello et al. (2006) but are similar to results of Guuroh et al. (2018). Zerbo et al. (2016) studied the effects of climate using a climatic gradient from the Sahel to the southern‐Sudanian zone of Burkina Faso, while Guuroh et al. (2018) studied a climatic gradient from the guinea savanna zone in Ghana to the sudan savanna zone in Burkina Faso. The gradient studied by Guuroh et al. (2018) is more similar to the current study as it included more mesic sites than the study of Zerbo et al. (2016). However, our study extends the climatic gradient to include even more mesic sites than Guuroh et al. (2018). Our study found the opposite of Guuroh et al. (2018) in terms of the effect of moisture on gamma diversity, indicating that less mesic sites of the study gradient favor recruitment and growth of herbaceous species than the more mesic sites. This result may be related to other site conditions such as disturbances and soil conditions that promote herbaceous species abundance. For example, less mesic sites are known to experience higher frequency and likelihood of disturbances such as grazing and fire, which positively influence herbaceous species diversity (Zerbo et al. 2016; Blench and Sommer 1999; Hahn‐Hadjali et al. 2006). Our study supports this position, as we found a positive relationship between diversity (SR and H′) and disturbance agents—fire and grazing (Figure 5). We also observed that heavily disturbed poor soils tend to have higher diversity of herbaceous species than less disturbed rich soils, a condition which may have also accounted for this result.

The observed negative relationship between herbaceous diversity and rainfall may also be caused by local scale processes such as evolutionary history (Harrison and Grace 2007) and seed abundance/dispersal limitation (Pärtel and Zobel 2007; Zobel and Pärtel 2008). Dispersal limitation could lead to species rarity (Bruno 2002; Mabry 2004) or patch occupancy (Matlack 2005; Helm et al. 2006). Additionally, higher alpha diversity in arid areas (Table 5) might also be related to the species‐area relationship/sample size effect (Oksanen 1996; Gotelli and Colwell 2001). Aridity may decrease plant size; a mechanism that could result in increased alpha diversity in arid conditions compared to humid conditions, especially if a fixed plot size is used (Oksanen 1996).

**TABLE 5:** Mean species richness and Shannon–Wiener index of the herbaceous layer along the climatic gradient and land‐use types.

Land‐use/climate zone	Species richness			Shannon–Weiner Index		
	Dry	Intermediate	Wet	Dry	Intermediate	Wet
NPA	11.10 ± 0.75_a_^A^	6.20 ± 0.55_a_^B^	10.40 ± 0.96_a_^A^	1.65 ± 0.13_a_^Z^	1.07 ± 0.09_a_^X^	1.23 ± 0.13_a_^ZX^
PBA	7.70 ± 0.93_b_^A^	7.10 ± 0.66_a_^A^	2.70 ± 0.40_b_^B^	1.06 ± 0.07_b_^Z^	0.99 ± 0.13_a_^Z^	0.59 ± 0.14_b_^X^
PUA	9.00 ± 0.79_a_b^A^	8.50 ± 0.79_a_^A^	0.60 ± 0.31_b_^B^	1.17 ± 0.10_a_^Z^	1.29 ± 0.15_a_^Z^	0.11 ± 0.11_b_^Z^

*Note:* Means within the same column followed by the same subscript lowercase letter are not statistically different at alpha = 0.05. Also, means within the same row for each diversity variable followed by the same superscript capital letter are not statistically different at alpha = 0.05.

Abbreviations: NPA, non‐protected burnt area; PBA, protected burnt area; PUA, protected unburnt area.

The effect of land use on SR varied with ecological zone; NPA had the highest in the wet FSTZ and dry SSZ while PUA had the highest in the intermediate GSZ (Table 5). Disturbances due to fires, grazing, and other activities in the FSTZ which maintain high light levels in the understorey account for the higher SR in the NPA while in the dry SSZ, heterogeneity in soil characteristics (particularly soil moisture) in conjunction with land use pressures supported the relatively higher SR among land‐uses (especially in the NPA). This suggests that climate may modulate land‐use effects on herbaceous SR. The lack of properly delineated land‐use types in the savanna is also evident by the clearer distinction in SR within the FSTZ than the GSZ and SSZ. Therefore, both climate and local (plot‐level) drivers may directly influence herbaceous species diversity. The findings highlight the importance of the interactive effects of climate and land‐use variables on plant composition and diversity focusing on the two complementary layers in savannas of Ghana (Hahn‐Hadjali et al. 2006; Zerbo et al. 2016). Unlike trees, aridity was the most important driver of herbaceous diversity indicating that climate has direct effects on herbaceous species.

## 4 Conclusion

This study examined the predictors and drivers of species composition and diversity under the interactive effects of climate and land‐use conditions in the semi‐arid areas of Ghana. The marked differences in species composition and diversity between ecological zones imply that climate change will indeed have an effect on vegetation. The greater importance of land use (grazing pressure) on the woody layer diversity and climate variables for the herbaceous layer imply that both anthropogenic and ecological drivers play significant roles in influencing species diversity. It must be acknowledged that a large‐scale driver such as climate tends to have long‐term indirect effects on species diversity through its effects on soil properties, grazing, and fire pressures. It can be deduced that appropriate land management strategies can potentially mitigate the negative effects of climate change on vegetation. Examples of such land management practices within the context of the study include protection against fires and grazing, prescribed fires, moisture and soil fertility management, and avoiding degradation due to land‐use conversions, etc. The study provides useful insights on the drivers of woody and herbaceous plant species diversity and could serve as a guide for resource managers and conservationists. The findings highlight that appropriate land management strategies may be used to mitigate potential negative effects of climate change and land‐use practices. Our results are useful for ecosystem management within the context of ongoing climate change as our study uses a space‐for‐time substitution approach to gain some insights about climate change effects on vegetation composition and diversity. We acknowledge that due to erratic climate and species shifts as well as land‐use management, species diversity and composition projections based on space‐for‐time substitution can be limited in the short term. Hence, long‐term monitoring of these sites is recommended.

## Author Contributions

**Reginald T. Guuroh:** conceptualization (lead), data curation (equal), formal analysis (equal), funding acquisition (lead), investigation (lead), methodology (equal), project administration (lead), resources (equal), software (equal), supervision (lead), validation (equal), visualization (equal), writing – original draft (lead), writing – review and editing (equal). **Bertrand F. Nero:** data curation (supporting), formal analysis (supporting), methodology (supporting), resources (supporting), software (supporting), writing – review and editing (supporting). **Fousseni Folega:** formal analysis (supporting), methodology (supporting), writing – review and editing (supporting). **Kwame A. Oduro:** conceptualization (supporting), data curation (supporting), methodology (supporting), project administration (supporting), resources (supporting), writing – review and editing (supporting). **Fred Kalanzi:** data curation (supporting), methodology (supporting), writing – review and editing (supporting). **Gloria K. Adeyiga:** data curation (supporting), resources (supporting), writing – review and editing (supporting). **Adu‐Gyamfi Asamoah:** data curation (supporting), project administration (supporting), writing – review and editing (supporting). **Mark Appiah:** data curation (supporting), project administration (supporting), writing – review and editing (supporting). **Miracle Obeng:** data curation (supporting), writing – review and editing (supporting). **E. Amponsah:** data curation (supporting), writing – review and editing (supporting).

## Conflicts of Interest

The authors declare no conflicts of interest.

## Supporting information


Data S1.

## Data Availability

The data for this manuscript has been uploaded as Supporting Information in CSV format (named as ‘Data‐Guuroh_et al.’ and Meta‐Data_Guuroh et al.). The R script used for analysis has also been uploaded and named R script_Guuroh et al.

## Appendix 1 Results of Principal Component Analysis (PCA) for Climate Variables. High Factor Loadings (≥|0.8|) on Principal Components (PC) With Eigenvalues > 1 are Shown in Bold. Variables Selected as Predictors for Diversity and Aboveground Biomass are Also in Bold

**Table:**

Variables	PC1	PC2	PC3	PC4	PC5	PC6	PC7	PC8	PC9	PC10	PC11
Mean annual temperature	0.9	0.36	−0.12	0.23	−0.04	0	0.01	0.01	0.02	0	0
Isothermality	−0.44	−0.48	0.19	−0.74	−0.01	0	0	0	0	0	0
**Minimum Temperature of the Coldest Month**	−0.17	**−0.90**	0.34	−0.21	0.02	−0.01	0.05	0.03	0	0.01	0
**Mean Temperature of the Wettest Quarter**	−0.05	−0.14	**0.98**	−0.09	0.01	0	0	0	0	0	0
Mean Temperature of the Driest Quarter	0.91	0.35	0.14	0.12	0.15	0.02	0.01	0	0	0	0
Mean Temperature of the Warmest Quarter	0.83	0.44	−0.1	0.31	−0.04	−0.04	0.01	0.02	0	0	0.01
**Mean Temperature of the Coldest Quarter**	**0.95**	0.22	−0.12	0.14	−0.06	0.01	−0.01	−0.01	−0.01	0	0
Annual Precipitation	−0.71	−0.52	0.26	−0.38	0.04	0.12	0	0	0	0	0
Precipitation of the Wettest Month	0.53	0.81	0.02	0.22	0.02	0.02	−0.02	0.06	0	0	0
Precipitation Seasonality	0.62	0.72	−0.1	0.28	0	−0.08	0.02	0.01	0	0.02	0
Precipitation of the Wettest Quarter	0.54	0.82	−0.04	0.17	0.02	−0.01	0.1	−0.01	0	0	0

## Appendix 2 Results of Principal Component Analysis (PCA) for (a) Soil Chemical Properties and (b) Soil Physical Properties. High Factor Loadings (≥|0.8|) on Principal Components (PC) With Eigenvalues > 1 are Shown in Bold. Variables Selected as Predictors for Diversity and Aboveground Biomass are Also in Bold

**Table:**

Variable	PC1	PC2	PC3	PC4	PC5	PC6	PC7	PC8	PC9	PC10	PC11
(a) Soil chemical properties											
**Soil acidity**	0.14	−0.13	0.06	−0.15	**0.95**	0.16	0.01	0	0.01	0	0
Organic carbon	0.93	0.2	0.15	0.14	0.01	0.15	−0.13	−0.02	−0.07	0.04	0
Total nitrogen	0.92	0.06	0.09	0.06	0.13	0.13	0.33	0	0.02	0	0
**Organic matter**	**0.95**	0.1	0.21	−0.09	0.05	0.12	−0.15	0.02	−0.02	−0.04	0
Calcium	0.79	0.32	0.27	−0.29	0.19	0.15	0.07	0.06	0.2	0	0
**Magnesium**	0.29	**0.86**	0.32	0.05	−0.03	0.11	0	0.21	0.04	0	0
**Sodium**	−0.01	0.24	−0.2	**0.93**	−0.16	0.12	0.01	0	−0.01	0	0
**Base saturation**	0.3	0.18	0.16	0.13	0.2	**0.89**	0.01	0	0	0	0
Sand content	−0.23	−0.41	−0.87	0.05	0	−0.12	0	0.04	0	0	0
**Silt content**	0.2	0	**0.95**	−0.19	0.08	0.08	0	0.04	0.01	0	0
Clay content	0.12	0.92	0.05	0.27	−0.15	0.12	0	−0.16	−0.02	0	0
(b) Soil physical properties											
Fine soil cover	−0.69	−0.37	−0.49	−0.38	−0.09	0					
**Fine gravel cover**	**0.96**	0.07	0.23	0.14	0.01	0					
Medium gravel cover	0.61	0.27	0.34	0.66	0.04	0					
**Coarse gravel cover**	0.34	0.19	**0.9**	0.18	0.04	0					
Stones cover	0.14	0.77	0.38	0.36	0.33	0					
**Boulders cover**	0.14	**0.98**	0.09	0.07	−0.06	0					

## Appendix 3 Correlation Between Selected Variables. Values Shown in Bold Indicate a High Correlation (>|0.7|) Between Variables; in These Cases, the Term That Performed Better in Single‐Variable Models was Chosen for Subsequent Statistical Analyses

**Table:**

Predictor	A	MTCQ	MiTCM	MTWeQ	Fire	GP	OM	Mg	Silt	Na	pH	Basesatu
A	1.0	1.0	**−0.7**	−0.3	0.1	0.6	−0.4	0.1	0.2	−0.1	−0.1	−0.4
MTCQ	**1.0**	1.0	−0.4	−0.2	0.1	0.5	−0.5	0.1	0.1	0.1	−0.1	−0.4
MiTCM	−**0.7**	−0.4	1.0	0.5	0.2	−0.3	0.2	0.0	−0.1	0.3	0.1	0.3
MTWeQ	−0.3	−0.2	0.5	1.0	0.2	0.1	0.1	0.1	−0.4	0.1	0.0	0.0
Fire	0.1	0.1	0.2	0.2	1.0	0.6	−0.3	0.0	−0.3	0.0	0.3	−0.2
GP	0.6	0.5	−0.3	0.1	0.6	1.0	−0.3	0.1	−0.2	−0.1	0.2	−0.3
OM	−0.4	−0.5	0.2	0.1	−0.3	−0.3	1.0	0.4	0.4	−0.1	0.2	0.4
Mg	0.1	0.1	0.0	0.1	0.0	0.1	0.4	1.0	0.4	0.2	−0.1	0.4
Silt	0.2	0.1	−0.1	−0.4	−0.3	−0.2	0.4	0.4	1.0	−0.4	0.2	0.3
Na	−0.1	0.1	0.3	0.1	0.0	−0.1	−0.1	0.2	−0.4	1.0	−0.3	0.2
pH	−0.1	−0.1	0.1	0.0	0.3	0.2	0.2	−0.1	0.2	−0.3	1.0	0.4
Basesatu	−0.4	−0.4	0.3	0.0	−0.2	−0.3	0.4	0.4	0.3	0.2	0.4	1.0

Abbreviations: A, aridity; Basesatu, soil base saturation; Fire, fire occurrence; GP, grazing pressure; Mg, soil magnesium; MiTCM, minimum temperature of the coldest month; MTCQ, mean temperature of the coldest quarter; MTWeQ, Mean Temperature of the Wettest Quarter; OM, organic matter; pH, soil acidity; Silt, soil silt content.

## Appendix 4 List of Woody Species Recorded in the Woody Layer

**Table:**

Species name	Family	Abundance
*Acacia angulata* Desv.	Fabaceae	2
*Acacia dudgeoni* Holland	Fabaceae	1
*Acacia gourmaensis* A. Chev.	Fabaceae	6
*Acacia seyal* Delile	Fabaceae	4
*Acacia sieberiana* DC.	Fabaceae	5
*Adansonia digitata* L.	Malvaceae	1
*Afraegle paniculata* (Schumach. & Thonn.) Engl.	Rutaceae	4
*Afrormosia laxiflora* Harms	Fabaceae	7
*Afzelia africana* Pers.	Fabaceae	10
*Aidia genipiflora* (DC.) Dandy	Rubiaceae	7
*Albizia coriaria* Oliv.	Fabaceae	2
*Albizia ferruginea* (Guill. & Perr.) Benth.	Fabaceae	1
*Albizia zygia* (DC.) J.F.Macbr.	Fabaceae	5
*Allophylus africanus* P.Beauv.	Sapindaceae	6
*Anacardium occidentale* L.	Anacardiaceae	1
*Aningeria robusta* (A.Chev.) Aubrév. & Pellegr.	Sapotaceae	2
*Annona senegalensis* Pers.	Annonaceae	1
*Anogeissus leiocarpa* (DC.) Guill. & Perr.	Combretaceae	30
*Antiaris toxicaria* Lesch.	Moraceae	7
*Azadirachta Indica* A.Juss.	Meliaceae	5
*Balanites aegyptiaca* (L.) Delile	Zygophyllaceae	6
*Blighia sapida* K.D.Koenig	Sapindaceae	1
*Blighia unijugata* Baker	Sapindaceae	1
*Bombax costatum* Pellegr. & Vuillet	Malvaceae	1
*Bauhinia variegata* L.	Fabaceae	1
*Boswellia dalzielii* Hutch.	Burseraceae	1
*Bridelia ferruginea* Benth.	Euphorbiaceae	4
*Bridelia grandis* ex Hutch.	Euphorbiaceae	1
*Bridelia micrantha* (Hochst.) Baill.	Euphorbiaceae	5
*Broussonetia papyrifera* (L.) L'Hér. ex Vent.	Moraceae	5
*Burkea africana* Hook.	Fabaceae	16
*Carapa procera* DC.	Meliaceae	8
*Cassia sieberiana* DC.	Fabaceae	1
*Ceiba pentandra* (L.) Gaertn.	Malvaceae	9
*Celtis philippensis* Blanco	Cannabaceae	1
*Chassalia afzelii* (Hiern) K.Schum.	Rubiaceae	2
*Cola gigantea* A.Chev.	Malvaceae	3
*Cola nitida* (Vent.) Schott & Endl.	Malvaceae	3
*Combretum adenogonium* Steud. ex A.Rich.	Combretaceae	24
*Combretum glutinosum* Perr. ex DC.	Combretaceae	6
*Combretum molle* R.Br. ex G. Don	Combretaceae	9
*Combretum nigricans* Lepr. ex Guill. & Perr.	Combretaceae	2
*Combretum sp*	Combretaceae	8
*Cordia millenii* Baker	Boraginaceae	1
*Crossopteryx febrifuga* (Afzel. ex G. Don) Benth.	Rubiaceae	12
*Cussonia barteri* Seem.	Araliaceae	3
*Daniella ogea* Rolfe	Fabaceae	1
*Daniellia oliveri* (Rolfe) Hutch. & Dalziel	Fabaceae	18
*Detarium microcarpum* Guill. & Perr.	Fabaceae	12
*Dichapetalum guineense* (DC.) Keay	Dichapetalaceae	1
*Dictyandra arborescens* Welw. ex Hook. f.	Rubiaceae	2
*Deinbollia grandifolia* Hook. f.	Sapindaceae	1
*Diospyros mespiliformis* Hochst. ex A.DC.	Ebenaceae	14
*Diospyros monbuttensis* Gürke	Ebenaceae	1
*Diospyros viridicans* Hiern	Ebenaceae	2
*Discoglypremna caloneura* (Pax) Prain	Euphorbiaceae	1
*Erythrophleum africanum* (Benth.) Harms	Fabaceae	3
*Erythrophleum ivorense* A.Chev.	Fabaceae	4
*Faidherbia albida* (Delile) A.Chev.	Fabaceae	1
*Ficus capensis* Thunb.	Moraceae	3
*Ficus exasperata* Vahl	Moraceae	5
*Ficus sp*	Moraceae	1
*Ficus vogelii* (Miq.) Miq.	Moraceae	2
*Gardenia aqualla* Stapf & Hutch.	Rubiaceae	5
*Gmelina arborea* Roxb.	Lamiaceae	1
*Grewia mollis* Juss.	Malvaceae	1
*Grewia occidentalis* L.	Malvaceae	1
*Hannoa undulata* (Guill. & Perr.) Planch.	Simaroubaceae	3
*Holarrhena floribunda* (G. Don) T.Durand & Schinz	Apocynaceae	10
*Hymenocardia acida* Tul.	Euphorbiaceae	6
*Isoberlinia doka* Craib & Stapf	Fabaceae	4
*Khaya grandifoliola* C.DC.	Meliaceae	2
*Khaya ivorensis* A.Chev.	Meliaceae	5
*Khaya senegalensis* (Desv.) A.Juss.	Meliaceae	3
*Lannea acida* A.Rich.	Anacardiaceae	26
*Lannea kerstingii* Engl. & K.Krause	Anacardiaceae	1
*Lannea nigritana* (Scott‐Elliot) Keay	Anacardiaceae	3
*Lecaniodiscus cupanioides* Planch. ex Benth.	Sapindaceae	11
*Lonchocarpus sericeus* (Poir.) DC.	Fabaceae	6
*Lophira lanceolata* Tiegh. ex Keay	Ochnaceae	5
*Mangifera indica* L.	Anacardiaceae	1
*Maranthes corymbosa* Blume	Chrysobalanaceae	2
*Margaritaria discoidea* (Baill.) G.L.Webster	Euphorbiaceae	5
*Maytenus senegalensis* (Lam.) Exell	Celastraceae	7
*Milicia excelsa* (Welw.) C.C.Berg	Moraceae	2
*Monotes kerstingii* Gilg	Dipterocarpaceae	3
*Monodora tenuifolia* Benth.	Annonaceae	2
*Morinda lucida* Benth.	Rubiaceae	3
*Morus mesozygia* Stapf	Moraceae	4
*Myrianthus arboreus* P.Beauv.	Urticaceae	1
*Nauclea latifolia* Sm.	Rubiaceae	5
*Newbouldia laevis* (P.Beauv.) Seem.	Bignoniaceae	4
*Ouratea reticulata* (P. Beauv.) Engl.	Ochnaceae	3
*Oxyanthus unilocularis* Hiern	Rubiaceae	5
*Parinari polyandra* Benth.	Chrysobalanaceae	2
*Parinari curatellifolia* Planch. ex Benth.	Chrysobalanaceae	8
*Parkia bicolor* A.Chev.	Fabaceae	1
*Parkia biglobosa* (Jacq.) G. Don	Fabaceae	8
*Pericopsis laxiflora* (Baker) Meeuwen	Fabaceae	3
*Phyllanthus reticulatus* Poir.	Euphorbiaceae	2
*Piliostigma thonningii* (Schum.) Milne‐Redh.	Fabaceae	19
*Piper guineense* Schumach. & Thonn.	Piperaceae	4
*Piptadeniastrum africanum* (Hook.f.) Brenan	Fabaceae	4
*Pouteria alnifolia* (Baker) Roberty	Sapotaceae	10
*Pseudocedrela kotschyi* (Schweinf.) Harms	Meliaceae	6
*Pteleopsis suberosa* Engl. & Diels	Combretaceae	1
*Pterocarpus erinaceus* Poir.	Fabaceae	16
*Pterocarpus macrocarpus* Kurz	Fabaceae	1
*Pycnanthus angolensis* (Welw.) Warb.	Myristicaceae	2
*Rauvolfia vomitoria* Afzel.	Apocynaceae	2
*Sclerocarya birrea* (A.Rich.) Hochst.	Anacardiaceae	3
*Securidaca longipedunculata* Fresen.	Polygalaceae	1
*Senna siamea* (Lam.) H.S.Irwin & Barneby	Fabaceae	3
*Spathodea campanulata* P.Beauv.	Bignoniaceae	8
*Stereospermum kunthianum* Cham.	Bignoniaceae	2
*Sterculia setigera* Delile	Malvaceae	5
*Sterculia tragacantha* Lindl.	Malvaceae	5
*Strophanthus hispidus* DC.	Apocynaceae	2
*Strychnos innocua* Delile	Loganiaceae	2
*Strychnos spinosa*	Loganiaceae	2
*Terminalia avicennioides* Guill. & Perr.	Combretaceae	42
*Terminalia catappa* L.	Combretaceae	1
*Terminalia glaucescens* Planch. ex Benth.	Combretaceae	7
*Terminalia microcarpa* F.Muell.	Combretaceae	4
*Terminalia mollis* M.A.Lawson	Combretaceae	1
*Tetrapleura tetraptera* (Schum. & Thonn.) Taub.	Fabaceae	2
*Syzygium guineense* (Willd.) DC.	Myrtaceae	2
*Trichilia monadelpha* (Thonn.) J.J.de Wilde	Meliaceae	1
*Trichilia prieuriana* A.Juss.	Meliaceae	6
*Vernonia conferta* Benth.	Asteraceae	1
*Vitellaria paradoxa* C.F.Gaertn.	Sapotaceae	44
*Vitex doniana* Sweet	Lamiaceae	1
*Vitex simplicifolia* Oliv.	Lamiaceae	2
*Vitex* spp.	Lamiaceae	1
*Ximenia americana* L.	Olacaceae	1
*Xylia evansii* Hutch.	Fabaceae	1
*Zanthoxylum leprieurii* Guill. & Perr.	Rutaceae	1
*Ziziphus mauritiana* Lam.	Rhamnaceae	1

## Appendix 5 ANOVA Results for the Woody Layer

**Table:**

	Species richness				
	Df	Sum Sq	Mean Sq	F value	Pr(>F)
Climate zone	2.000	660.100	330.000	40.950	0.000
Land‐use	2.000	487.300	243.600	30.230	0.000
Climate zone × Land‐use	4.000	289.900	72.500	8.990	0.000
Residuals	81.000	652.900	8.100		
	Shannon Weiner Index				
Climate zone	2.000	11.829	5.915	25.417	0.000
Land‐use	2.000	8.457	4.229	18.172	0.000
Climate zone × Land‐use	4.000	3.540	0.885	3.803	0.007
Residuals	81.000	18.849	0.233		

*Note:* Signif. codes: 0 ‘***’ 0.001 ‘**’ 0.01 ‘*’ 0.05 ‘.’ 0.1 ‘’ 1.

## Appendix 6 Percent Variance Explained by Linear Mixed‐Effects Models for (a) the Woody Layer, (b) the Herbaceous Layer. Total Variance Explained is Based on Conditional *R* ^2^ Values, Variance Explained by Fixed‐Effects is Based on Marginal *R* ^2^ Values; for Random‐Effects (Variance Explained by Site Alone), It Is Based on Conditional *R* ^2^ Values Minus Marginal *R* ^2^ Values. Residuals Quantify Unexplained Variance for the Respective Ecosystem Service

**Table:**

(a) woody layer				
Response variable	Marginal *R* ^2^ (MR^2^) (%)	Conditional R^2^ (CR^2^) (%)	Site effects CR^2^ minus MR^2^	Residual
Species richness model	69.28	71.07	1.79	28.93
Shannon–Wiener Index model	62.31	76.82	14.51	23.18
(b) Herbaceous layer				
Response variable	Marginal R^2^ (MR^2^) (%)	Conditional R^2^ (CR^2^) (%)	Site effects CR^2^ minus MR^2^	Residual
Species richness model	56.70	78.50	21.80	21.50
Shannon–Wiener Index model	49.89	60.15	10.26	39.85

## Appendix 7 Direct and Indirect Effects of Various Variables Used in the SEM Model for Woody and Herbaceous Layers

**Table:**

Variables	Woody layer		Herbaceous layer	
	Direct	Indirect	Direct	Indirect
Climate—Land‐use	−0.37	0.00	−0.38	0.00
Climate—Soil	0.28	0.05	0.24	0.06
Climate—Diversity	0.38	0.19	−0.33	−0.18
Inter—Soil	−0.32	0.00	−0.29	0.00
Inter—Diversity	−0.14	0.01	0.25	0.06
Land‐use—Soil	−0.14	0.00	−0.15	0.00
Land‐use—Diversity	−0.54	0.00	0.32	0.03
Soil—Diversity	−0.02	0.00	−0.22	0.00

## Appendix 8 List of Species Recorded in the Herbaceous Layer

**Table:**

Species name	Family	Abundance
*Acacia dudgeoni* Holland	Fabaceae	1
*Achyranthes aspera* L.	Amaranthaceae	4
*Aeschynomene indica* L.	Fabaceae	1
*Ageratum conyzoides* (L.) L.	Asteraceae	1
*Alysicarpus ovalifolius* (Schum.) Leonard	Fabaceae	12
*Andropogon gayanus* Kunth	Poaceae	29
*Andropogon chinensis* (Nees) Merr.	Poaceae	11
*Anogeissus leiocarpa* (DC.) Guill. & Perr.	Combretaceae	3
*Aristida kerstingii* Pilg.	Poaceae	13
*Aspilia helianthoides* (Schumach. & Thonn.) Oliv. & Hiern	Asteraceae	5
*Aspilia paludosa* Berhaut	Asteraceae	12
*Brachiaria jubata* (Fig. & De Not.) Stapf	Poaceae	2
*Brachiaria lata* (Schumach.) C.E.Hubb.	Poaceae	15
Burkea africana Hook.	Fabaceae	2
*Cassia mimosoides*	Fabaceae	17
*Chloris pilosa* Schumach. & Thonn.	Poaceae	4
*Chasmopodium caudatum* (Hack.) Stapf	Poaceae	1
*Chromolaena odorata*	Asteraceae	6
*Cochlospermum tinctorium* Perrier ex A.Rich.	Bixaceae	3
*Commelina nigritana* Benth.	Commelinaceae	9
*Corchorus tridens* L.	Malvaceae	1
*Crinum paludosum* Verd.	Amaryllidaceae	5
*Crotalaria retusa* L.	Fabaceae	14
*Ctenium elegans* Kunth	Poaceae	3
*Cucumis melo* L.	Cucurbitaceae	1
*Cymbopogon giganteus* Chiov.	Poaceae	1
*Cyperus amabilis* Vahl	Cyperaceae	4
*Cyperus tenuiculmis* Boeckeler	Cyperaceae	2
*Dactyloctenium aegyptium* (L.) Willd.	Poaceae	4
*Daniellia oliveri* (Rolfe) Hutch. & Dalziel	Fabaceae	5
*Digitaria horizontalis* Willd.	Poaceae	23
*Dioscorea togoensis* R.Knuth	Dioscoreaceae	1
*Echinochloa colona* (L.) Link	Poaceae	13
*Eragrostis pilosa* (L.) P.Beauv.	Poaceae	3
*Euclasta condylotricha* (Steud.) Stapf	Poaceae	1
*Euphorbia hirta* L.	Euphorbiaceae	3
*Evolvulus alsinoides* (L.) L.	Convolvulaceae	1
*Ficus exasperata* Vahl	Moraceae	1
*Fimbristylis ferruginea* (L.) Vahl	Cyperaceae	14
*Fimbristylis littoralis* Gaudich.	Cyperaceae	2
*Gladiolus gregarius* Welw. ex Baker	Iridaceae	1
*Heteropogon contortus* (L.) P.Beauv. ex Roem. & Schult.	Poaceae	9
*Hibiscus asper* Hook.f.	Malvaceae	4
*Hoslundia opposita* Vahl	Lamiaceae	1
*Hygrophila micrantha* (Nees) T.Anderson	Acanthaceae	7
*Hyparrhenia cyanescens* (Stapf) Stapf	Poaceae	1
*Hyptis spicigera* Lam.	Lamiaceae	7
*Hyptis suaveolens* (L.) Poit.	Lamiaceae	4
*Indigofera bracteolata* DC.	Fabaceae	1
*Indigofera dendroides* Jacq.	Fabaceae	2
*Indigofera macrocalyx* Guill. & Perr.	Fabaceae	14
*Indigofera tinctoria* L.	Fabaceae	4
*Ipomoea eriocarpa* R. Br.	Convolvulaceae	1
Kaempferia aethiopica (Schweinf.) Ridl.	Zingiberaceae	1
*Kyllinga pumila* Michx.	Cyperaceae	10
Lannea acida A.Rich.	Anacardiaceae	1
*Lepidagathis anobrya* Nees	Acanthaceae	3
Mariscus squarrosus (L.) C.B.Clarke	Cyperaceae	2
*Mimosa pudica* L.	Fabaceae	8
*Monechma ciliatum* (Jacq.) Milne‐Redh.	Acanthaceae	10
*Nelsonia canescens* (Lam.) Spreng.	Acanthaceae	7
*Pandiaka heudelotii* (Moq.) Benth. & Hook.f. ex B.D.Jacks.	Amaranthaceae	31
*Paspalum orbiculare* G.Forst.	Poaceae	1
*Piliostigma thonningii* (Schum.) Milne‐Redh.	Fabaceae	1
*Polygala arenaria* Willd.	Polygalaceae	5
*Polycarpaea eriantha* Hochst. ex A.Rich.	Caryophyllaceae	1
*Polycarpaea linearifolia* (DC.) DC.	Caryophyllaceae	1
*Pseudocedrela kotschyi* (Schweinf.) Harms	Meliaceae	1
*Pteleopsis suberosa* Engl. & Diels	Combretaceae	1
*Pterocarpus erinaceus* Poir.	Fabaceae	2
*Rottboellia exaltata* (L.) L.f.	Poaceae	4
*Scoparia dulcis* L.	Plantaginaceae	9
*Setaria pumila* (Poir.) Roem. & Schult.	Poaceae	12
*Sida acuta* Burm.f.	Malvaceae	3
*Sida alba* L.	Malvaceae	5
*Sida linifolia* Juss. ex Cav.	Malvaceae	2
*Sida rhombifolia* L.	Malvaceae	1
*Sida urens* L.	Malvaceae	1
*Spermacoce filifolia* (Schumach. & Thonn.) J.‐P. Lebrun & Stork	Rubiaceae	27
*Spermacoce stachydea* DC.	Rubiaceae	50
*Sporobolus festivus* Hochst. ex A.Rich.	Poaceae	2
*Sporobolus pectinellus* Mez	Poaceae	2
*Sporobolus pyramidalis* P.Beauv.	Poaceae	6
*Striga hermonthica* (Delile) Benth.	Orobanchaceae	2
*Stylosanthes erecta* P.Beauv.	Fabaceae	6
*Tephrosia bracteolata* Guill. & Perr.	Fabaceae	44
*Tephrosia pedicellata* Baker	Fabaceae	12
*Terminalia avicennioides* Guill. & Perr.	Combretaceae	5
*Tridax procumbens* (L.) L.	Asteraceae	2
*Tripogon minimus* (A.Rich.) Hochst. ex Steud.	Poaceae	8
*Vigna racemosa* (G. Don) Hutch. & Dalziel	Fabaceae	2
*Vitex doniana* Sweet	Lamiaceae	2
*Vitellaria paradoxa* C.F.Gaertn.	Sapotaceae	2
*Waltheria indica* L.	Malvaceae	12
*Zornia glochidiata* DC.	Fabaceae	4
*Afama	—	1
*Droben	—	2
*Mamfohuma	—	1
*Mate	—	2
*Nankakete	—	3
*Ntwantini	—	2
*Nyame wu na ma wu	—	1
*Nyeremonyini	—	2

## Appendix 9 ANOVA Results for the Herbaceous Layer

**Table:**

	Species richness				
	Df	Sum Sq	Mean Sq	*F* value	Pr(>F)
Climate zone	2.000	333.800	166.900	32.740	0.000
Land‐use	2.000	218.400	109.200	21.420	0.000
Climate zone × Land‐use	4.000	399.800	99.950	19.610	0.000
Residuals	81.000	412.900	5.100		
Shannon Weiner Index					
Climate zone	2.000	6.697	3.348	24.322	0.000
Land‐use	2.000	4.087	2.043	14.842	0.000
Climate zone × Land‐use	4.000	4.754	1.189	8.633	0.000
Residuals	81.000	11.151	0.138		

*Note:* Signif. codes: 0 ‘***’ 0.001 ‘**’ 0.01 ‘*’ 0.05 ‘.’ 0.1 ‘’ 1.
